# Procedural Sedation Using Two Different Proportions of Ketamine-Propofol Combination in Short Gynecological Procedures: A Randomized Controlled Trial

**DOI:** 10.7759/cureus.21393

**Published:** 2022-01-18

**Authors:** Pulak P Padhi, Sirisha Yeeda, Laba K Nayak, Saswati Das

**Affiliations:** 1 Department of Anesthesia, Kalinga Institute of Medical Sciences, Kalinga Institute of Industrial Technology (KIIT) Deemed to be University, Bhubaneswar, IND; 2 Department of Anesthesia, Manipal Hospitals, Guntur, IND

**Keywords:** sedation, ketofol, analgesia, procedural sedation, propofol, ketamine

## Abstract

Background: Procedural sedation with a combination of propofol and ketamine for short-duration surgeries is a convenient technique of anesthesia as it has a faster recovery avoiding the side effects of general anesthesia. The aim of this study was to compare the sedative and analgesic effects of two different proportions of ketamine and propofol combination in patients undergoing short gynecological procedures.

Methods: A randomized double-blind study was conducted in 140 patients posted for elective gynecological procedures with a duration equal to or less than 30 minutes. After premedication of all participants, sedation was induced with bolus administration (0.1 mL/kg) of the study drugs to achieve desired Ramsay sedation score (RSS) of 6, followed by infusion at 0.3 mL/kg/h (Group A, ketamine:propofol in the ratio of 1:4 and Group B, ketamine:propofol in the ratio of 1:2). The adequacy of sedation, volume of drug to induce the patient, time to achieve desired RSS, time for first bolus dose, the total volume of the drugs, hemodynamic variables, awakening time, and side effects were observed.

Results: The incidence of movement of lower extremities was found to be significantly lower in the higher concentration ketamine group (Group B, P - 0.028). The volume of a drug for induction and the duration to reach RSS of 6 were significantly lower in Group B with P-values of 0.002 and <0.001, respectively. Hemodynamic variables, awakening time, and side effects were not statistically significant between the two groups.

Conclusion: Ketamine-propofol combination in the ratio 1:2 provides better sedation and analgesia with no increased side-effects compared to ketamine-propofol in the ratio 1:4 for short outpatient gynecological procedures.

## Introduction

Short gynecological procedures are mostly done as outpatient day-case procedures where the patients are discharged on the same day of admission after the intended procedure. Procedural sedation is a convenient technique of anesthesia for these procedures which provides adequate anesthetic depth and hemodynamic stability with early recovery and minimum adverse eﬀects in the recovery period.

Various drugs have been tried to achieve the goals of day-case procedures done under sedation. Since no single drug can provide all the requirements of procedural sedation, different drugs are used in varying combinations to provide balanced anesthesia, that is, amnesia, hypnosis, and analgesia [1]. Ketamine-propofol combination has been used in varying proportions for procedural sedation in a variety of procedures both in outpatient and emergency department settings with good results. The opposing hemodynamic and respiratory effects of each drug may enhance the use of this combination thereby increasing both safety and efficacy and allowing a reduction in the dose of propofol required to achieve sedation and decrease the need for supplemental opioid analgesics [2,3]. The combination of these two drugs has been used in many clinical situations, with better hemodynamic stability, minimal respiratory depression, better analgesia, and recovery than each agent alone [4-13]. The effectiveness of the two agents, ketamine and propofol in combination mixed in a single syringe has demonstrated efficacy in operating and ambulatory settings in varying proportions with varying results but the ideal proportion has not been established yet. We conducted this study using 1:2 and 1:4 ratios of ketamine propofol combination in short gynecological procedures for providing procedural sedation. The primary objective of the study was to compare the adequacy of sedation and analgesia provided by two different ratios of ketamine propofol combination in patients undergoing short gynecological outpatient procedures. The secondary objectives were to compare the hemodynamic variables, airway intervention if any, time for awakening, and the incidence of side effects between the two groups. We hypothesized that ketofol in the ratio of 1:2 would provide better analgesia and sedation compared to ketofol in the ratio of 1:4.

## Materials and methods

After the approval of the Institutional Ethics Committee (#KIMS/KIIT/IEC/156/2018) and written informed consent, this randomized double-blind study was conducted between September 2019 and January 2021, in 140 female patients in the age group of 18-60 years, belonging to American Society of Anesthesiologists (ASA) physical status I or II, undergoing elective short gynecological daycare procedures lasting less than 30 minutes. Patients with a history of allergy to study drugs, obstructive sleep apnea, and behavioral problems were excluded from the study. The trial was registered with CTRI with registration number CTRI/2019/08/020808.

Patients were asked to fast as per the standard Nil Per Oral guidelines. Patients were randomly assigned to one of the two groups using computer-based randomization. An intravenous catheter was secured on the dorsum of the non-dominant hand in the preoperative waiting room and premedication of intravenous Glycopyrrolate 0.2 mg and Midazolam 1 mg were given to all patients 10 minutes prior to induction. After shifting the patients to the operating room, standard ASA monitors were attached which included 5 lead electrocardiograms, pulse oximeter, and non-invasive blood pressure. Oxygen was delivered to all patients by a face mask at 6 L/min. Sedation was induced by bolus intravenous administration of 0.1 mL/kg of the study drug.

**Figure 1:**
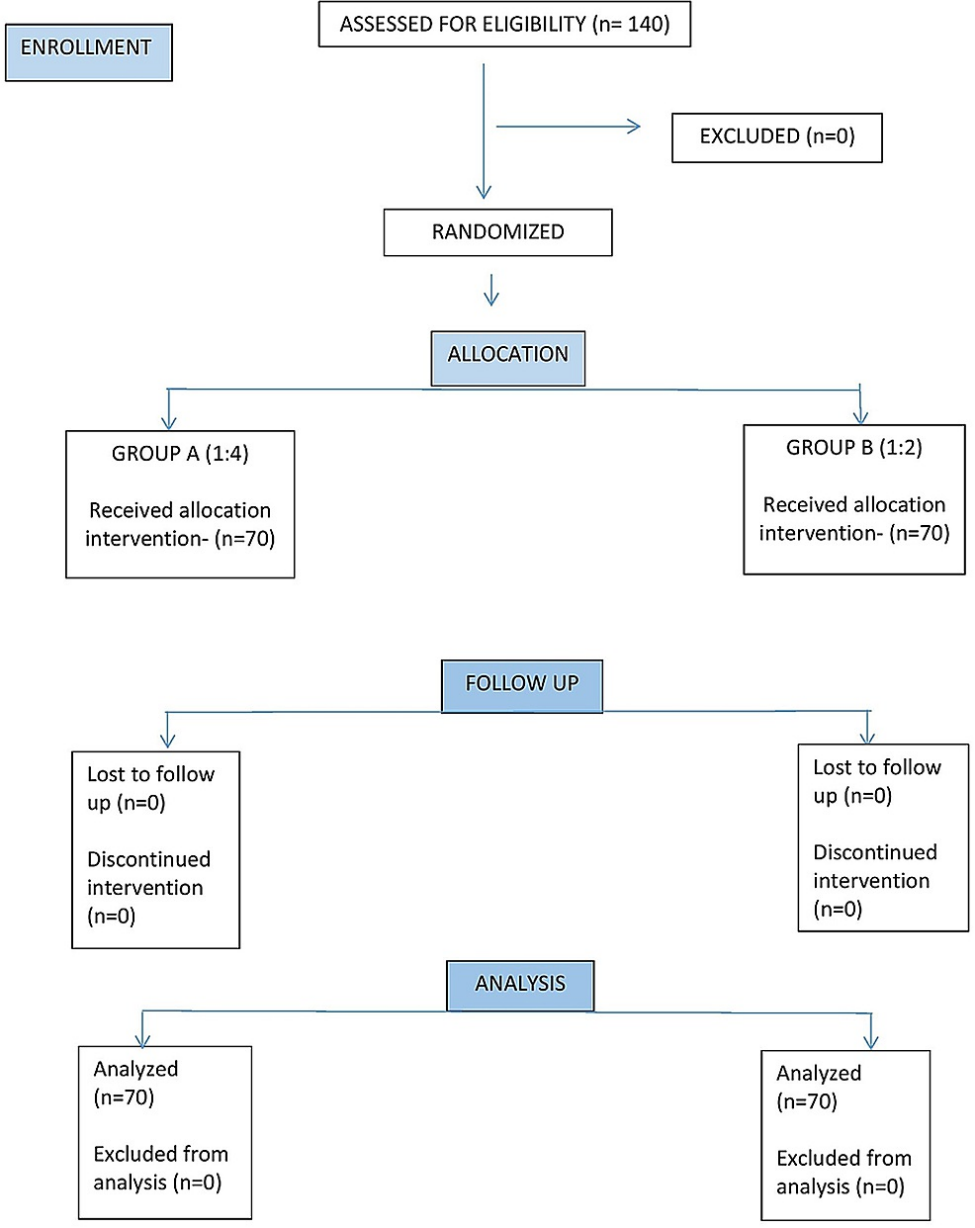
Consort flow chart

Group A: 1:4 ratio of ketamine-propofol combination (1 mL of 50 mg/mL Ketamine added to 20 mL of 1% propofol and 4 mL of 5% Dextrose to make a total volume of 25 mL) and Group B: 1:2 ratio of the ketamine-propofol combination (2 mL of 50 mg/mL Ketamine added to 20 mL of 1% Propofol and 3 mL of 5% Dextrose to make a total volume of 25 mL) (Figure 1).

After sedation was induced with bolus administration of the study drugs, it was maintained with an infusion of the study drug at 0.3 mL/kg/hr. The drugs used to induce and maintain anesthesia were prepared in the same syringes by an anesthetist not involved in the study. Ramsay Sedation Score (RSS) of 6 was considered satisfactory and the surgeon was allowed to proceed with the surgery. If the patient did not achieve the desired RSS, a 2 mL bolus of the study drug was administered.

The parameters like systolic blood pressure, diastolic blood pressure, mean arterial blood pressure, heart rate, respiratory rate, oxygen saturation, and depth of sedation were assessed at baseline (before injecting the study drug), and every two minutes till the end of the procedure. The observations were recorded by an independent researcher who was blinded to the study group. End-tidal carbon dioxide (EtCO_2_) was monitored continuously by a side stream sampling line inserted into the facial mask. If apnea occurred, as assessed clinically or by capnography trace, or if the peripheral oxygen saturation (SPO_2_) was ≤ 96%, a jaw thrust maneuver was performed by the anesthetist. If effective ventilation was not achieved after the initial maneuver, bag-mask ventilation was performed.

If there was an incidence of movement in the lower extremities during the procedure, a 2 mL bolus of the study drug was administered. Induction, maintenance, and delivery of bolus doses were done using a single syringe pump. After the procedure, the patient was transferred to the Post Anesthesia Care Unit (PACU) and monitored until they met discharge criteria assessed by the Modified Aldrete Score of ≥ 9.

The primary objective of the study was to compare the adequacy of sedation in both groups. This was assessed by the incidence of movement in the lower extremities. The secondary objectives were to compare the volume of drug required for induction, duration to reach RSS of 6, time taken for administration of first bolus dose, a total number of bolus doses administered, the total volume of drug used, hemodynamic variables, time for awakening (defined as the time from the discontinuation of infusion at the end of surgery till the patient responds to verbal commands), airway intervention if any, postoperative nausea and vomiting (PONV), recovery agitation and recall of intraoperative events. PONV if any was treated with Ondansetron 4 mg IV.

Oh et al. [14] observed the prevalence of movement in lower extremities was 32.5% and 10% in 1:3 ketofol group and 2:3 ketofol combination, respectively. Based on this at a 5% level of significance and 90% power, the sample size was calculated as 67 in each group. To adjust for any dropouts, 70 patients were recruited in each group.

Statistical analysis was performed using SPSS® version 20.0 (SPSS, Chicago, IL, USA). For continuous variables, the data were presented as mean ± SD using Student’s t-test, and the categorical variables were presented as frequency and percentage. The Chi-square test or Fisher exact test was used to check the association between the two different groups and a P-value of ≤0.05 was considered to be statistically significant.

## Results

A total of 140 patients were enrolled in the study, 70 in each group. The baseline characteristics such as age, weight, height, and body mass index (BMI) were similar in the two groups (Table 1).

**Table 1:** Baseline characteristics The data are represented as mean± standard deviation (SD) and analyzed using unpaired student’s t-test. A P-value of ≤0.05 is considered statistically significant.

Baseline characteristics	Group A (Ketofol 1:4)	Group B (Ketofol 1:2)	P-value
Age (years)	34.54±8.07	35.91±8.92	0.342
Height (feet)	5.10±0.31	5.08±0.277	0.625
Weight (kg)	59.93±7.19	58.24±5.95	0.133
BMI	24.42±2.63	23.89±2.32	0.211
Duration of procedure (minutes)	11.71±4.867	12.10±4.682	0.633

**Figure 2:**
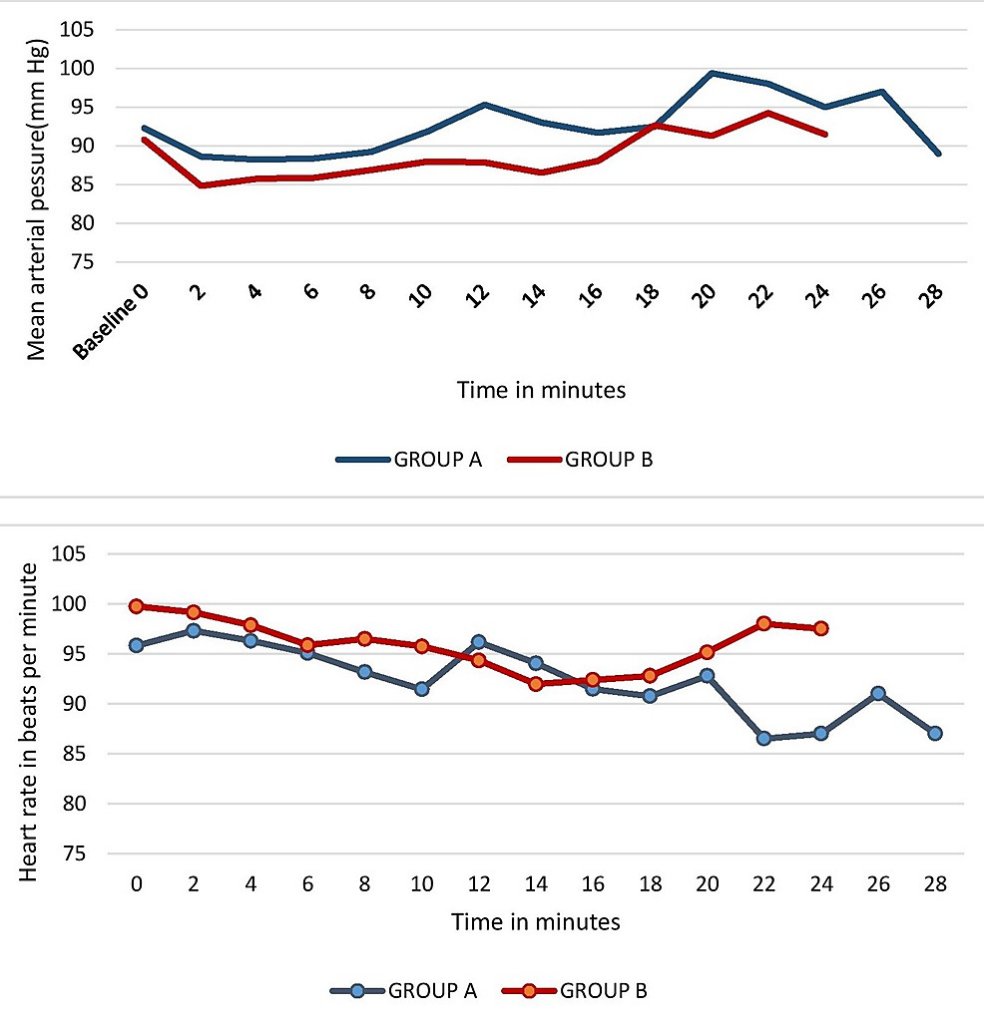
Comparison of mean arterial pressure and heart rate at two-minute intervals

The incidence of movement in lower extremities that correlates with the number of patients receiving bolus doses was significantly lower in Group B (30,42.9%) compared to group A (43, 61.4%) with a P-value of 0.028. The number of bolus doses and the time for administration of the first bolus dose were not statistically different between the two groups. The time taken to reach an RSS of 6 was significantly lower (P<0.001)) in Group B compared to Group A. Also, the volume of drugs for induction was significantly lower in Group B compared to Group A with a P-value of 0.002. The total volume of drug used, total duration of the procedure, and awakening time were not statistically different between the two groups (Table 2). There was no statistically significant difference in the blood pressure readings, heart rate, and respiratory rate (RR) in the two groups (Figure 2).

**Table 2:** Primary and secondary objectives The data are represented as a number (percent) or as mean± standard deviation and analyzed using Chi-square test or unpaired student’s t-test as appropriate. A P-value of ≤0.05 is considered statistically significant.

Objectives	Group A (Ketofol 1:4)	Group B (Ketofol 1:2)	P-value
Incidence of movement	43 (61.4)	30 (42.9)	0.028
Volume of drug for induction (mL)	8.89±1.584	8.09±1.462	0.002
Duration to achieve Ramsay sedation score of 6 (seconds)	56.27±8.339	50±10.621	<0.001
Time for 1st bolus dose (seconds)	5.93±2.939	7.03±3.045	0.125
Total number of bolus doses	1.04±1.055	0.90±1.426	0.502
Total volume of drug used (mL)	14.37±3.972	13.34±4.596	0.159
Awakening time (minutes)	2.24±1.837	2.31±1.556	0.804

The incidence of PONV in Group A and group B was 17.1% and 14.3%, respectively, and this difference was statistically insignificant (P=0.642). Recovery agitation was seen in only one of the patients in Group B whereas none of the patients in Group A had recovery agitation (P=1.000). No patient in either group had a recall of intra-operative events (Table 3).

**Table 3:** Incidence of side-effects Data presented as number (percent) and analyzed using the Chi-square test. A P-value of ≤0.05 is considered statistically significant. PONV - postoperative nausea and vomiting

Side effects	Group A (Ketofol 1:4)	Group B (Ketofol 1:2)	P-value
Recall of intra-operative events	0	0	
Recovery agitation	0 (0.0)	1 (14)	1.000
Airway intervention	5 (7.1)	3 (4.3)	0.718
PONV	12 (17.1)	10 (14.3)	0.642

## Discussion

Procedural sedation for short surgical procedures is most commonly carried out with ketamine or propofol in addition to opioids and benzodiazepines. Ketamine commonly produces emergence delirium and vomiting along with an increase in heart rate and blood pressure in the routine induction dose. Propofol at induction dose can result in a severe fall in blood pressure and does not have any analgesic properties. The aim of anesthesia in short outpatient gynecological procedures is to reduce the patient's anxiety, ensure adequate sedation and analgesia during the procedure and facilitate early recovery with minimal side effects for an early discharge. The combination of propofol and ketamine produces more stable hemodynamic conditions than ketamine or propofol used individually. The combination of ketamine and propofol in different proportions is being used for procedural sedation because of the increased analgesic eﬀect of ketamine and reduction of the side eﬀects of propofol. In the present study, we compared the sedative and analgesic eﬀects, hemodynamics and respiratory changes, the requirement of amount of anesthetic solutions, recovery times, and complications of two different ratios of ketamine propofol combination in 140 patients undergoing daycare gynecological procedures.

In this study, we found the incidence of movement in lower extremities was significantly lower in the 1:2 ketofol group compared to 1:4 ketofol group. A similar result was reported in a study conducted by Oh et al. [14] with an aim to reduce patient movement in loop electrosurgical excision procedure. They found that the incidence of adduction motion in lower extremities was significantly lower in patients receiving higher ketofol concentrations. Similar results were found in a trial studying different doses of ketamine with propofol in patients undergoing breast biopsy procedures [15]. The incidence of movement correlated to the number of patients needing bolus doses of the study drug as it was administered when the patient responded to surgical stimulus. In our study, the number of patients requiring bolus doses was significantly higher in group A compared to Group B. This was in concurrence with studies conducted by other authors [15-17]. This was due to the higher concentration of ketamine in group B providing better analgesia.

The volume of drug used during induction to achieve desired RSS of 6 was significantly lower in Group B compared to Group A. Using a lower dose of propofol in the ketofol mixture helps avoid the problems with excess propofol use like hemodynamic instability and the need for airway intervention. This is practically beneficial to both patients and clinicians.

The time required for the patients to reach an RSS of 6 after the induction dose was found to be significantly higher in group A compared to group B in our study. In the study conducted by Badrinath et al. [15], they found no difference in the time required to achieve the desired Observer Assessment of Alertness score. This could be possibly due to the different concentrations of ketamine in the ketofol groups studied.

Our study showed that the total number of bolus doses given when there was a response to the surgical stimulus was insignificant in both groups. A similar observation was made in studies conducted by other authors [15,18]. However, in a study by Oh et al. [14], a statistically significant difference was observed. This could be due to the lower concentration of ketamine in the ketofol mixture needing more boluses.

The total volume of drugs used was not statistically different in our study although it was observed to be lower in the higher ketamine group (Group B). Similarly, in the study concluded by Miner et al. [19] the total sedative bolus dose requirement was higher in the lower ketamine concentration group. Our study did not find any statistically significant difference in the hemodynamic parameters measured throughout the procedure which aligned with findings from previous studies [15-17].

The need for airway intervention in our study was insignificant between the two groups, which were similar to a few studies conducted earlier [16,18]. However, in the studies conducted by other authors [14,17], there was a statistically significant difference in the need for airway intervention in the groups receiving higher ketamine in the ketofol mixture. The difference in the findings could be due to the deep level of sedation with a higher dose of ketamine in the ketofol mixture which led to impaired breathing and increased need for airway support.

The awakening time in both groups was statistically and clinically insignificant in our study. Similar results were found in a study conducted by Miner et al. [19]. The recovery time in our study was significantly longer in the group with higher ketamine concentration. This was also demonstrated in studies conducted by other authors [16,17,19].

We noticed recovery agitation in one patient in the higher ketamine group and none in the lower ketamine group. It was transient and the patient did not require any restraining or use of opioids or benzodiazepines. Similar results with no difference in the incidence of recovery agitation were found in other studies [14,16]. However, studies conducted by other authors [17,19] found a higher incidence of recovery agitation in the group receiving 1:1 ketofol compared to the other groups with lower ketamine concentrations. This could be due to the higher concentration of ketamine in 1:1 ketofol group.

The incidence of PONV in PACU in our study was found to be statistically insignificant. Similar results were found in studies conducted by previous authors [15-18]. No patients in either group experienced recall of intraoperative events. Similar results were documented by other authors [14,15].

The limitations of our study were that the depth of sedation was assessed by RSS. The use of bispectral index monitoring could have been done for a better assessment of depth of anesthesia during procedural sedation. In addition, a specific scoring system to measure the analgesic component of the patients undergoing procedures was lacking in our study. Future studies can be carried out taking these into consideration.

## Conclusions

Procedural sedation for short gynecological procedures can be safely and effectively carried out using a 1:2 ratio of ketamine propofol combination. Based on the findings of our study, we state that the use of ketamine-propofol combination in the ratio 1:2 provides better sedation and analgesia with fewer patients needing additional boluses compared to ketamine-propofol in the ratio 1:4 for short outpatient gynecological procedures.
